# Can super resolution via deep learning improve classification accuracy in dental radiography?

**DOI:** 10.1093/dmfr/twaf029

**Published:** 2025-04-15

**Authors:** Berrin Çelik, Mahsa Mikaeili, Mehmet Z Genç, Mahmut E Çelik

**Affiliations:** Oral and Maxillofacial Radiology Department, Faculty of Dentistry, Ankara Yıldırım Beyazıt University, Ankara, 06010, Turkey; Biomedical Calibration and Research Center (BIYOKAM), Gazi University Hospital, Gazi University, Ankara, 06560, Turkey; Mechatronics Engineering Department, Faculty of Engineering and Natural Sciences, İstanbul Okan University Tuzla Campus, Istanbul 34959, Turkey; Electrical Electronics Engineering Department, Faculty of Engineering, Gazi University, Ankara, 06570, Turkey; Biomedical Calibration and Research Center (BIYOKAM), Gazi University Hospital, Gazi University, Ankara, 06560, Turkey; Electrical Electronics Engineering Department, Faculty of Engineering, Gazi University, Ankara, 06570, Turkey

**Keywords:** radiographic magnification, deep learning, dentistry, panoramic, X-ray, artificial intelligence

## Abstract

**Objectives:**

Deep learning-driven super resolution (SR) aims to enhance the quality and resolution of images, offering potential benefits in dental imaging. Although extensive research has focused on deep learning based dental classification tasks, the impact of applying SR techniques on classification remains underexplored. This study seeks to address this gap by evaluating and comparing the performance of deep learning classification models on dental images with and without SR enhancement.

**Methods:**

An open-source dental image dataset was utilized to investigate the impact of SR on image classification performance. SR was applied by 2 models with a scaling ratio of 2 and 4, while classification was performed by 4 deep learning models. Performances were evaluated by well-accepted metrics like structural similarity index (SSIM), peak signal-to-noise ratio (PSNR), accuracy, recall, precision, and F1 score. The effect of SR on classification performance is interpreted through 2 different approaches.

**Results:**

Two SR models yielded average SSIM and PSNR values of 0.904 and 36.71 for increasing resolution with 2 scaling ratios. Average accuracy and F-1 score for the classification trained and tested with 2 SR-generated images were 0.859 and 0.873. In the first of the comparisons carried out with 2 different approaches, it was observed that the accuracy increased in at least half of the cases (8 out of 16) when different models and scaling ratios were considered, while in the second approach, SR showed a significantly higher performance for almost all cases (12 out of 16).

**Conclusion:**

This study demonstrated that the classification with SR-generated images significantly improved outcomes.

**Advances in knowledge:**

For the first time, the classification performance of dental radiographs with improved resolution by SR has been investigated. Significant performance improvement was observed compared to the case without SR.

## Introduction

In recent years, deep learning has emerged as a transformative force across various disciplines, including dentistry. Dental image analysis via deep learning has applied almost every subfield of dentistry, from orthodontics and endodontics to periodontics and oral surgery.^1–4^ These advanced computational techniques have enhanced the accuracy and efficiency of diagnosing dental conditions by enabling automated detection and classification of complex patterns in radiographic images, cone-beam computed tomography (CBCT), and intraoral radiographs. The increasing adoption of deep learning tools reflects a paradigm shift towards more data-driven and precise dental care, offering the potential to improve patient outcomes and streamline clinical workflows. The potential applications of artificial intelligence (AI) in dentistry, which can technically be categorized as classification, detection, and segmentation tasks, are vast, especially in automated image analysis. Its integration into dentistry represents a significant advancement with profound implications for clinical practice and patient outcomes. Leveraging sophisticated machine learning algorithms and computational techniques, AI has emerged as a transformative tool for improving various aspects of dental care, ranging from diagnosis and treatment planning to patient management and outcome prediction.

As an emerging research field, AI has the potential to significantly impact the quality of patient care by increasing the resolution and quality of dental images. There are studies in which the resolution of radiography images is successfully increased with AI models, ie, deep learning. The concept is called as super resolution (SR). SR is a set of techniques in computer vision that aim to enhance the resolution of an image or a sequence of images. The primary goal is to reconstruct a high-resolution (HR) image from one or more low-resolution (LR) images, effectively increasing the pixel count and improving the visual quality and detail. From engineering perspective, SR provides an ability to improve image quality without the need for hardware upgrades. From clinical perspective, it can be recovered rich features from a low-quality image taken with the current gadgets by utilizing deep learning-based SR algorithms, resulting HR images for the best diagnosis that is generally difficult and expensive as it calls for expensive, specialized equipment, skilled personnel, and which frequently results in operational delays.

SR can improve dental imaging, leading to more accurate diagnoses and better treatment planning. This not only aids in accurate diagnosis but also facilitates the development of more precise treatment plans, ultimately improving patient outcomes. Providing details of anatomical landmarks and pathologies are crucial and can provide some advantages which ultimately leads to safe, effective and predictable dental treatment.

It has been previously shown that SR method can be used to increase the resolution of radiographs. Some studies are based on medical image resolution enhancement of ultrasound and magnetic resonance imaging.^1^^,^^5–9^ In dentistry, although there are limited works which attempt to enhance resolution of dental radiographs, the effect of radiographs generated by SR on a classification problem has not been evaluated before. Previous studies briefly covered image resolution enhancement capability of SR techniques based on deep learning. Hatvani et al^10^ applied SR to enhance image resolution of 2-D CBCT, resulting in superior findings compared to reconstruction based methods. Another study by Hatvani et al^11^ again was towards developing computationally efficient techniques with comparatively less data that uses 3-D CBCT for resolution enhancement. Rytky et al^12^ applied SR to medical images including teeth images to address limited features in very small size area regarding tissue microstructure. Moran applied SR to increase image resolution of periapical images with peak signal-to-noise ratio (PSNR), structural similarity index (SSIM) metrics.^13^ Visual analysis reported better edge details and less blurring. Johnson et al^14^ replaced transposed convolution with resized convolution and utilized patch-by-patch sliding window approach. They attempted to enhance CBCT resolution with a factor of 4 using limited set of data such as knee tissue from cadavers and teeth from patients. Comparison of their results with conventional methods including image processing and interpolation demonstrated that deep learning model was superior in super resolving CBCT images. Li et al^15^ applied deep learning to predict mandibular third molar extraction difficulty. They used pre-trained ResNet-152 structure on the ImageNet for transfer learning of panoramic images. Wang et al^16^ used SwinIR network for SR of dental computed tomography images incorporated by the Self-Calibrated Convolutions Network (SCNet) for feature extraction, the cross-shaped windows, Self-Attention Transformer structure for context information acquisition, and the integration of the efficient channel attention module for local cross-channel interaction and model convergence acceleration. Kong et al^17^ applied SR to overcome the limitation of traditional image classification when dealing with large number of implants. Çelik et al^18^ utilized 4 different SR models to increase panoramic radiograph resolution.

This study aims to investigate the effect of SR concept, which is used to increase the resolution with deep learning, on the classification of dental images. Dental images from an open source dataset were classified both with and without SR, and the effect of increasing the resolution was compared. Four different classes of tooth images, namely incisor, canine, premolar, and molar, were manually cut from panoramic images. Totally, 6400 images, 1600 from each class, were used in the study. Four different deep learning models, ResNet, VGG, DenseNet, and EfficientNet, were used for classification, while Efficient Subpixel Super Resolution (ESPCN) and Super-Resolution Convolutional Neural Network (SRCNN) models were used for SR with scaling factors (SFs) of 2 and 4. The performance of each task was evaluated by using widely-recognized quantitative evaluation metrics. The performance of the classification task was evaluated by accuracy, recall, precision, and F1 score, while the SR task was measured by SSIM and PSNR.

## Methods

This section includes explanations on data preparation, scaling, the applied techniques, and evaluation metrics.

### Data preparation and scaling

**Figure 1.**
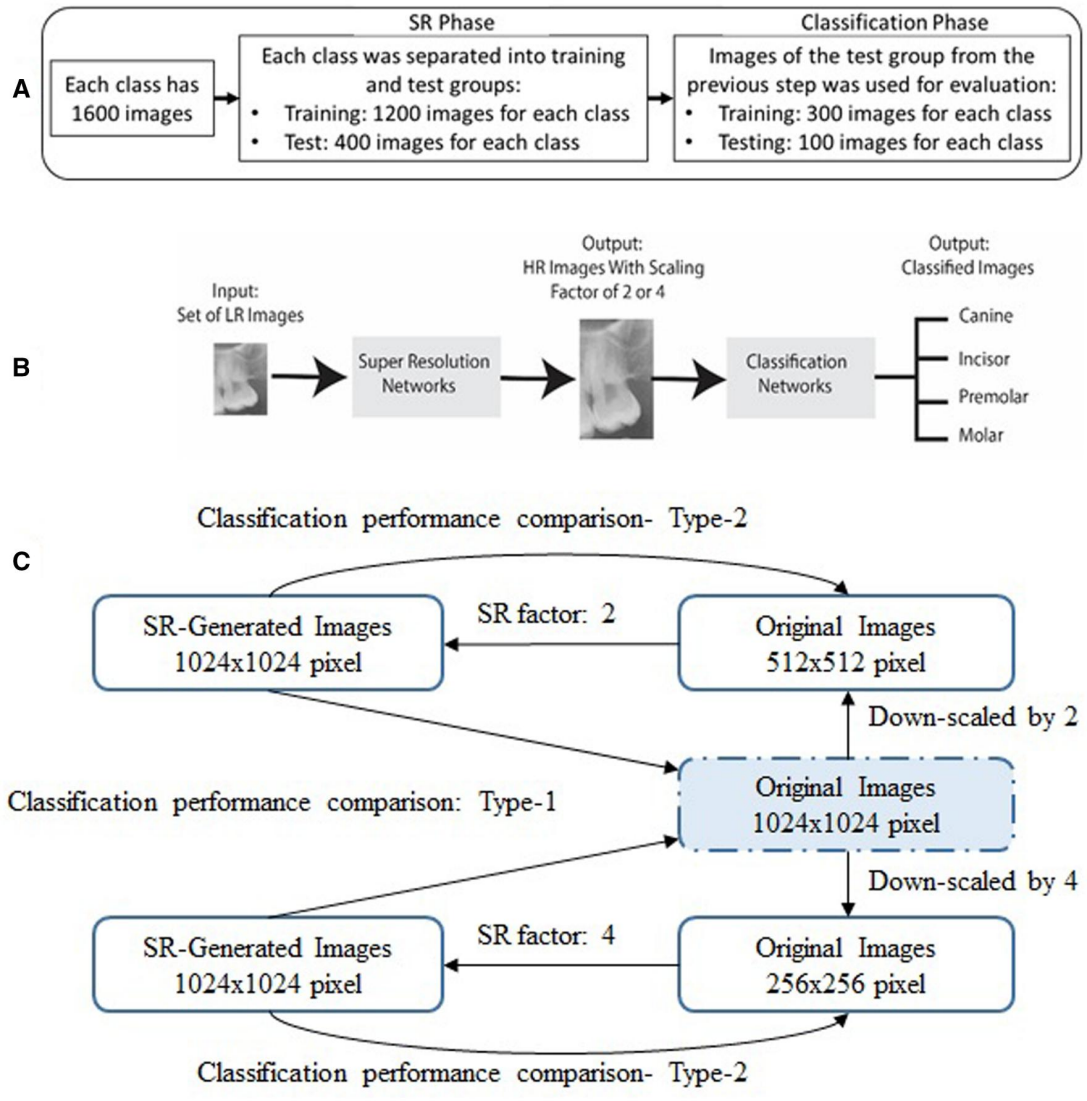
(A) Separation of data for SR and classification tasks, (B) block diagram of applied method, (C) 2 different approaches while comparing classification performances with and without super resolution. Abbreviations: HR = high resolution; LR = low resolution; SR = super resolution.

In this study, open access dataset of dental panoramic radiographic images from The Tufts Dental Database is utilized.^19^ Inclusion criteria were determined as teeth without any dental anomalies and root resorption for any reason. On the other hand, radiographs containing metallic artifacts, radiographs with dense artifacts that could not be clearly evaluated in the study area, and radiographs containing pathological formations due to cysts or tumours in the relevant area were excluded. Four types of teeth, which are incisor, canine, premolar, and molar, in the panoramic radiographs were manually cropped. Each class includes 1600 images. For each class, 1200 of them was used to train SR models while the remaining 400 was fed to SR models for test stage. For each class, original 400 images and SR generated 400 image-predictions were further analysed for classification purposes. For each class, 300 images were applied to training phase while the rest was used to test and compare classification models. For simplicity, separation of data was demonstrated in Figure 1A.

In SR stage, during training, input data were downscaled with 2 and 4 SFs respectively. In addition, in test stages, LR images (downscaled) are given to network, and its output is HR images with SF of 2 or 4. During classification, first, it performs on downscaled images for 2 and 4 SFs distinctively. Then, super-resolved images with SFs of 2 and 4 are forwarded to networks to perform classification tasks and ultimately to have a benchmark classification with original data. The experimentation was conducted using an Intel eighth generation i7 processor unit with 4 GHz and 32GB of RAM, along with an NVIDIA GeForce GTX 1050 graphics card.

### Deep learning models

The flowchart of this study is given in Figure 1B. LR images were applied to the deep learning-based SR methods which performs SR for 2 different SFs 2 and 4, respectively. After evaluating acquired results with 2 different evaluation metric, the obtained HR images were considered as inputs to the classification models.

There were 2 different deep learning based SR techniques and 4 different classification models.

The first SR model is ESPCN.^20^ This network is designed for the single-image SR task with capability of upscaling LR images directly to the HR images in a computationally effective manner. Main components of network are feature extraction, nonlinear mapping, and ultimately sub-pixel convolution. The network begins with a series of convolutional layers that extract hierarchical features from the LR input images. Hence, the network is capable to capture low- and high-level features. Following the feature extraction layers, there are additional nonlinear mapping layers, typically implemented using rectified linear unit (ReLU) activation functions. These layers introduce nonlinearity into the network, allowing it to learn complex mappings between LR and HR image spaces. The distinctive component of ESPCN is the sub-pixel convolutional layer. This layer is responsible for directly upscaling the LR feature maps to HR feature maps. It rearranges the feature maps in a way that effectively increases the spatial resolution.

Second SR model, SRCNN, extracts patches from LR images, and then the extracted patches are fed into a series of convolutional layers that serve to extract hierarchical features from the LR input patches.^21^ After nonlinear mapping by exploiting ReLU, reconstruction of HR images from the processed maps takes place. This is typically achieved using a single convolutional layer that aggregates the features learned by the network and produces the final HR output.

The third network is Efficient Super-Resolution Transformer (ESRT).^22^ It utilizes hybrid architecture that is composed of CNN and Transformer section and capable to handle small datasets for the task of SR. This network is composed of a lightweight and efficient deep learning model, leveraging Transformer-based self-attention mechanisms to capture long-range dependencies while maintaining computational efficiency. Unlike conventional CNN-based approaches, ESRT combines a shallow convolutional feature extractor with a Transformer encoder, allowing it to model both local and global contexts effectively. This hybrid design enables better texture restoration and detail enhancement while keeping the model lightweight. ESRT also incorporates multi-scale feature aggregation and efficient upsampling strategies to reconstruct HR images with improved sharpness and fidelity. Its ability to learn complex feature relationships makes it a powerful tool for SR tasks.

Downscaling is performed by bicubic interpolation for both downscaling ratios of 2 and 4. For the task of SR, we choose same hyper parameters for both networks. The SRCNN network has 3 convolution layers with ReLU as an activation function. The ESPCN network includes 3 convolutional layers plus one subpixel layer which makes a total of 4 layers. ReLU is applied as an activation function. The other parameters are as follows: patch size of 32, 800 epochs, learning rate of 0.0001, batch size of 128, Adam optimizer, and mean squared error (MSE) as a loss function.

On the other hand, the first classification model is Deep Residual Learning (ResNet).^23^ This network structure provides residual learning approach that facilitates the training of significantly deeper neural networks. The main concept of this network is employing shortcut connections that enable the training of very deep neural networks. It consists of residual blocks, each of which contain multiple convolutional layers. Each residual block includes shortcut connection that skips one or more layers. Hence, it allows the network to learn residual function with reference to the input to the block. This kind of connections enables the gradient to flow directly through the network without passing through multiple nonlinear activation functions, which in turn mitigates the vanishing gradient problem and facilitates the training of very deep networks. In order to increase the depth of the network, it involves stacking multiple residual blocks that enables learning of complex features at different levels.

The second classification model is VGG structure.^24^ Its architecture includes very small (3 × 3) convolution filters and nonlinear rectification layers to enhance the discriminative power of the decision function and to reduce the number of parameters compared to using larger convolutional filters. Hence, it reduces the number of parameters of the network while increasing its learning capacity and providing better generalization ability.

The third classification model is DenseNet.^25^ The architecture aims to maximize information flow between layers, which are connected to every other layer in a feed-forward fashion, in neural network. By connecting each layer directly to every other layer in a feed-forward manner, DenseNet addresses the vanishing gradient problem, strengthens feature propagation, and improves parameter efficiency. Unlike traditional convolutional networks where each layer is connected only to its subsequent layer, DenseNets have direct connections between all pairs of layers. This dense connectivity pattern allows each layer to receive feature maps from all preceding layers and pass its own feature maps to all subsequent layers and therefore enhances information flow and gradient propagation throughout the network.

The other classification model is EfficientNet.^26^ It provides compound scaling method for convolutional neural networks that balances network width, depth, and resolution to achieve better efficiency and accuracy. A compound scaling method uniformly scales all dimensions of network depth, width, and resolution using a simple yet highly effective compound coefficient. It balances the 3 dimensions of the network to achieve better performance. By scaling each dimension with a constant ratio, the network structure maintains a harmonious balance that leads to improved accuracy and efficiency.

The Vision Transformer (ViT) is an image classification model that adapts a Transformer-based architecture to process image patches.^27^ The input image is divided into fixed-size patches, which are then linearly transformed into embeddings. Positional encodings are incorporated to retain spatial information, and the resulting sequence of vectors is passed through a conventional Transformer encoder. For classification, a learnable “classification token” is appended to the sequence, following the standard Transformer methodology.

Considering parameters, ResNet has 23.8 million parameters, VGG has 139 million, DenseNet has 7.0 million, EfficientNet has 17.6 million, and ViT has 85.8 million. All models are trained with a batch size of 128, using the cross-entropy loss function over 50 epochs. The Adam optimizer is employed for all models, with a consistent learning rate of 1e−3.

### Evaluation metrics

Well-known evaluation metrics are used to evaluate each task, SR, and classification. There are 6 different evaluation metrics in total, 2 for SR and 4 for classification task.

First metric applied for evaluating SR task is SSIM method, which is a commonly used metric to evaluate the quality of photographs.^28^ Luminance, contrast, and structure are the 3 primary factors used to determine how similar 2 photos are to one another in this comprehensive reference measure. Structure evaluates the local brightness patterns between the photos to ascertain how similar or distinct they are, while contrast assesses the ranges that separate the brightest and darkest parts of the images. These 3 factors are multiplied to get SSIM.


(1) $$\mathrm{SSIM}=\frac{\left(2\mu_{x}\mu_{y}+c_{1})(2\sigma_{xy}+c_{2}\right)}{\left(\mu_{x}^{2}+\mu_{y}^{2}+c_{1})(\sigma_{x}^{2}+\sigma_{y}^{2}+c_{2}\right)}$$

where the x and y window averages are denoted by $\mu_{x}$ and $\mu_{y}$. Additionally, their corresponding variances are $\sigma_{x}^{2}$ and $\sigma_{y}^{2}$. The denominator cannot go to zero, which is the aim of $c_{1}$ and $c_{2}$.

The second evaluation metric for SR is PSNR.^28^ It is a widely used statistic to assess how well reconstructed pictures or videos are made. It provides a numerical evaluation of the reconstruction accuracy by comparing the original picture with a deformed or compressed version in order to gauge the quality of the image. The PSNR formula is given in eqn (2).


(2) $$\mathrm{PSNR}=10\times \log_{10}(\frac{{\left(2^{n}-1\right)}^{2}}{\mathrm{MSE}})$$

where *n* is the bit depth of the image; it equals to 255 for an 8-bit image as in our case (*n* = 8). The average squared difference between the original and deformed pictures is represented by MSE.

The Feature Similarity Index Method (FSIM) is a full-reference image quality metric that measures the similarity between 2 images based on their features.^28^ It primarily evaluates phase congruency (PC) and gradient magnitude (GM), which help assess image quality in a perceptually meaningful way. PC is resistant to changes in lighting and contrast, emphasizing image details in the frequency domain. FSIM is computed using a combination of PC and GM values. Equations (1) and (2) define the similarity scores based on these values. Here, ${PC}_{x}$,$G_{x}\;$and ${PC}_{y}$,$G_{y}\;$are extracted from the test image ($I_{x}$) and reference image ($I_{y}$), respectively. $G_{x}$ and $G_{y}$ represent the GMs, while $T_{x}$ and $T_{y}$ are constants that enhance stability and account for the dynamic range of GMs.


(3) $$S_{PC}=\frac{2{PC}_{x}{PC}_{y}+T_{x}}{{PC}_{x}^{2}+{PC}_{y}^{2}+T_{x}}$$

(4) $$S_{G}=\frac{2G_{x}G_{y}+T_{y}}{G_{x}^{2}+G_{y}^{2}+T_{y}}$$

The FSIM is determined by combining eqns (1) and (2) using eqn (8). In this equation, α and β are positive values that adjust the contributions of PC and GM, respectively. The final similarity score, $S_{L}$, is calculated as follows:


(5) $$S_{L}={\left[S_{PC}\right]}^{\alpha }.{\left[S_{G}\right]}^{\beta }$$

Multi-scale SSIM (MS-SSIM) is final evaluation metric.^29^ It extends the SSIM by analysing image luminance, contrast, and structural information at multiple scales. This multi-scale approach enhances accuracy compared to single-scale SSIM.


(6) $$\mathrm{MS}-\mathrm{SSIM}={\left[l_{m}(x,y)\right]}^{\alpha M}.\prod_{j=1}^{M}{\left[C_{j}(x,y)\right]}^{\beta j}.{\left[S_{j}(x,y)\right]}^{yj}$$

Here, MMM represents the lowest resolution, while *j* = 1 corresponds to the original resolution.

On the other hand, classification model performances are evaluated by accuracy, recall, precision, and F1 score. Accuracy is defined as the proportion of correctly classified instances among the total number of instances, as given in eqn (7).


(7) $$\mathrm{Accuracy}=\frac{\mathrm{TP}+\mathrm{TN}}{\mathrm{TP}+\mathrm{TN}+\mathrm{FP}+\mathrm{FN}}$$

Recall is defined as a proportion of true positive instances among all the actual positive instances, as given in eqn (8).


(8) $$\mathrm{Recall}=\frac{\mathrm{TP}}{\mathrm{TP}+\mathrm{FN}}$$

Precision refers to the proportion of true positive instances among all the instances that were classified as positive, as given in eqn (9).


(9) $$\mathrm{Precision}=\frac{\mathrm{TP}}{\mathrm{TP}+\mathrm{FP}}$$

Ultimately, F1 score gives the harmonic mean of precision and recall, providing a balance between the 2 metrics, presented in eqn (10).


(10) $$F1\;\mathrm{Score}=\frac{2.\;\mathrm{Precision}\;.\;\mathrm{Recall}}{\mathrm{Precision}+\mathrm{Recall}}=\frac{2TP}{2TP+\mathrm{Fp}+\mathrm{FN}}$$

While evaluating performances, intensity plots were also used. Pixel intensity refers to the brightness or colour value of a pixel in a digital image. It quantifies the amount of light or energy captured by the pixel and is used to represent the image’s visual information. An intensity plot provides both quantitative and spatial insights into pixel brightness values, making it a valuable tool for diagnosing, analysing, and preprocessing images in various fields. By offering a clear and detailed view of intensity variations, it enhances decision-making and improves the efficiency of image-related tasks.

When it comes to the comparison of classification performances with and without SR, it is necessary to clarify some points for those interested from all backgrounds.

In this study, it was mentioned that LR images were used for training deep learning based SR models by reducing the original image resolutions by a factor of 2 and 4 respectively. Thus, it will be possible to measure the performance of SR models by evaluating how similar the images generated by SR models are to real images. Otherwise, no evaluation metric can be calculated since we would not have a reference to evaluate how accurate the result is when we increase the resolution of the original resolution images by a factor of 2 or 4. Consequently, in this work, an image with a resolution higher than the image resolution of the original dataset is not produced, although it is technically possible.

Therefore, 2 different interpretations can be made when comparing the classification performance, as indicated in Figure 1C. The image resolution values presented in Figure 1C are examples of mathematically familiar numbers that are easy to imagine; the cropped image resolutions for tooth types are lower. The first one, Type-1, is to compare the classification results with the images produced by the SR models with the original image resolution, regardless of the SR factor, in accordance with this work plan (when an image with a resolution higher than the image resolution in the original dataset is not produced). Another approach represents the case when SR models are taught with higher resolution images and these technologies would be integrated into the system and is shown in Figure 1C as Type-2. For this case, the classification performance of the images whose resolution is increased by the SR factor can be compared with the classification performance of LR images.

## Results

The dataset was categorized into training and test sets. Each class, namely incisor, canine, premolar, and molar, had 1200 and 400 images for training and testing, respectively. The image resolutions were increased by a factor of 2 and 4 with the SR models, so the original image resolutions were downscaled by a factor of 2 and 4 beforehand and applied to the models. This makes it possible to measure the similarity of the resolution-enhanced images with the real images at the end of the SR process. Our evaluation relies on quantitative and qualitative methods. Quantitative evaluation is given in Table 1. Table 1 presents a comparative evaluation of 3 SR methods—ESPCN, SRCNN, and ESRT—at SFs of 2 and 4, across different dental structures (molar, premolar, canine, and incisor).

**Table 1.** Performance evaluation of SR stage.

	ESPCN, SF = 2				ESPCN, SF = 4			
	Molar	Premolar	Canine	Incisor	Molar	Premolar	Canine	Incisor
**SSIM**	0.9604	0.9521	0.9542	0.9583	0.8863	0.8650	0.8756	0.8819
**PSNR**	38.1476	37.2992	38.4833	39.5217	32.0118	31.7771	32.8418	33.7737
**FSIM**	0.9709	0.9662	0.9697	0.9724	0.9140	0.9034	0.9121	0.9164
**MS-SSIM**	0.9922	0.9909	0.9911	0.9919	0.9633	0.9589	0.9611	0.9633

	SRCNN, SF = 2				SRCNN, SF = 4			
	Molar	Premolar	Canine	Incisor	Molar	Premolar	Canine	Incisor
**SSIM**	0.9581	0.9491	0.9516	0.9558	0.8847	0.8631	0.8740	0.8800
**PSNR**	37.9131	37.0651	38.2644	39.2868	31.9014	31.6181	32.7658	33.6778
**FSIM**	0.9686	0.9634	0.9671	0.9699	0.9102	0.8990	0.9080	0.9121
**MS-SSIM**	0.9921	0.9907	0.9909	0.9917	0.9627	0.9583	0.9606	0.9629

	ESRT, SF = 2				ESRT, SF = 4			
	Molar	Premolar	Canine	Incisor	Molar	Premolar	Canine	Incisor
**SSIM**	0.9722	0.9449	0.9563	0.9496	0.8797	0.8216	0.8424	0.8414
**PSNR**	41.0493	37.9951	39.9361	39.7242	33.2746	31.0005	32.6802	32.8067
**FSIM**	0.9796	0.9610	0.9702	0.9651	0.9121	0.8803	0.8953	0.8982
**MS-SSIM**	0.9959	0.9908	0.9931	0.9911	0.9667	0.9436	0.9513	0.9453

Abbreviations: ESPCN = Efficient Subpixel Super Resolution; ESRT **=** Efficient Super-Resolution Transformer; SRCNN = Super-Resolution Convolutional Neural Network; SSIM = structural similarity index; PSNR = peak signal-to-noise ratio; FSIM = Feature Similarity Index Method; MS-SSIM = multi-scale structural similarity index.

The metrics used for evaluation include SSIM, PSNR, FSIM, and MS-SSIM, which assess image similarity, reconstruction quality, and perceptual accuracy. At SF = 2, ESRT demonstrates superior performance in terms of PSNR and FSIM, particularly for molars and canines, indicating that it preserves fine details and structure better than ESPCN and SRCNN. However, its SSIM scores for premolars and incisors are slightly lower than ESPCN, suggesting some variability in maintaining structural integrity across different tooth types. ESPCN consistently outperforms SRCNN in all metrics, highlighting its effectiveness in retaining image quality at moderate SR levels.

At SF = 4, a notable decline in performance is observed across all methods due to the increased difficulty of high upscaling. ESPCN and SRCNN exhibit similar trends, with ESPCN slightly outperforming SRCNN in all metrics, reinforcing its robustness. However, ESRT, which performed best at SF = 2, shows a more significant drop in SSIM and FSIM, particularly for premolars and incisors, suggesting that its effectiveness diminishes at higher SFs. Despite this, ESRT still maintains a relatively high PSNR compared to the other models, implying that it preserves overall brightness and contrast well but struggles with fine structural details. This suggests that while ESRT may be preferable for lower-scale SR tasks, ESPCN remains more stable across different SFs, making it a more reliable option for dental image SR when considering both moderate and high SFs.

**Figure 2.**
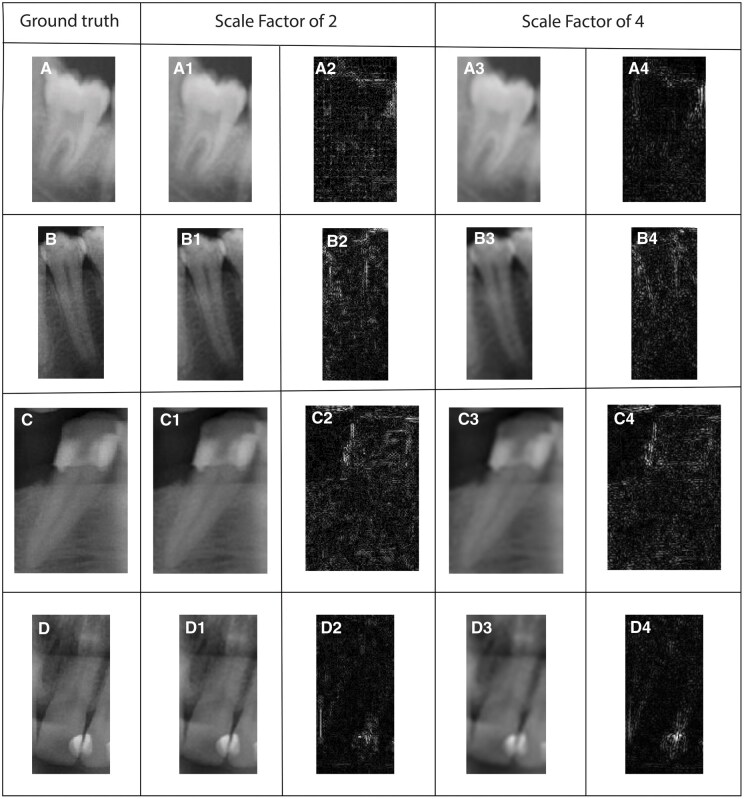
Results acquired from ESPCN network for both scaling factors 2 and 4. (A) Ground truth for molar class, (A1,2) SR result with scaling factor of 2 and difference map respectively for molar class, (A3,4) SR result with scaling factor of 4 and difference map respectively for molar class. (B) Ground truth for premolar class, (B1,2) SR result with scaling factor of 2 and difference map respectively for premolar class, (B3,4) SR result with scaling factor of 4 and difference map respectively for premolar class. (C) Ground truth for canine class, (C1,2) SR result with scaling factor of 2 and difference map respectively for canine class, (C3,4) SR result with scaling factor of 4 and difference map respectively for canine class. (D) Ground truth for incisor class, (D1,2) SR result with scaling factor of 2 and difference map respectively for incisor class, (D3,4) SR result with scaling factor of 4 and difference map respectively for incisor class.

**Figure 3.**
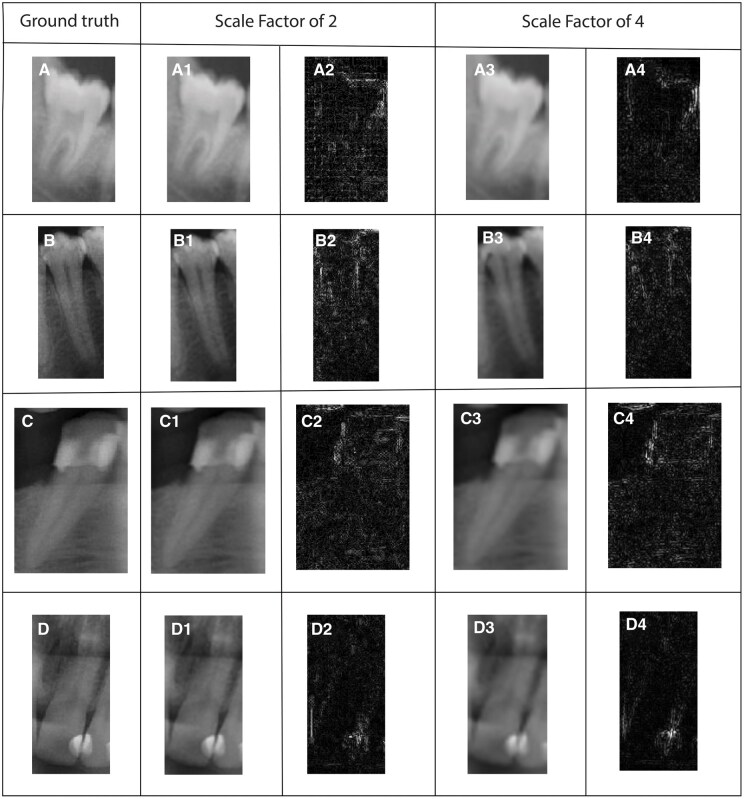
Results acquired from SRCNN network for both scaling factors 2 and 4. (A) Ground truth for molar class, (A1,2) SR result with scaling factor of 2 and difference map respectively for molar class, (A3,4) SR result with scaling factor of 4 and difference map respectively for molar class. (B) Ground truth for premolar class, (B1,2) SR result with scaling factor of 2 and difference map respectively for premolar class, (B3,4) SR result with scaling factor of 4 and difference map respectively for premolar class. (C) Ground truth for canine class, (C1,2) SR result with scaling factor of 2 and difference map respectively for canine class, (C3,4) SR result with scaling factor of 4 and difference map respectively for canine class. (D) Ground truth for incisor class, (D1,2) SR result with scaling factor of 2 and difference map respectively for incisor class, (D3,4) SR result with scaling factor of 4 and difference map respectively for incisor class.

**Figure 4.**
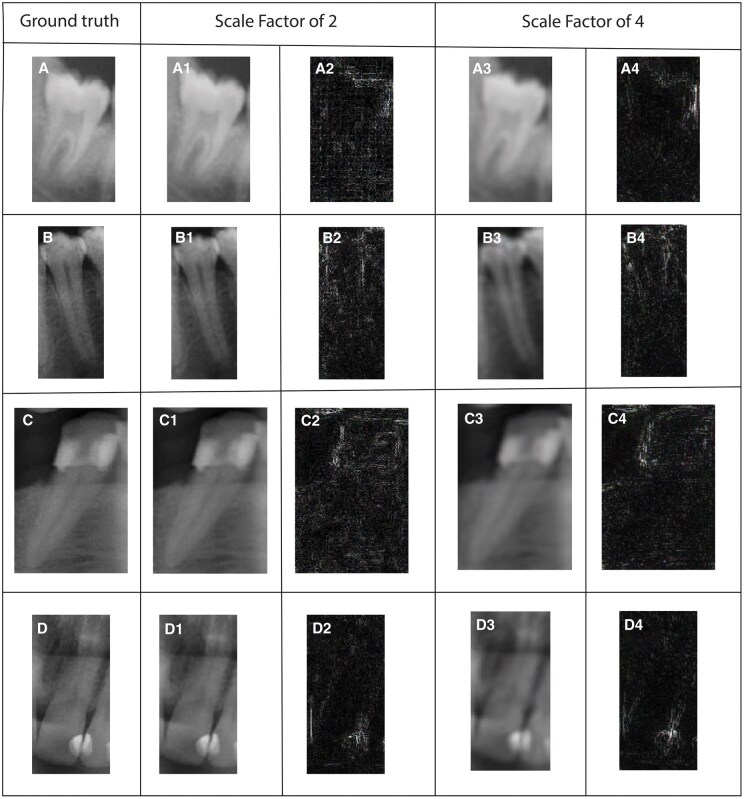
Results acquired from ESRT network for both scaling factors 2 and 4. (A) Ground truth for molar class, (A1,2) SR result with scaling factor of 2 and difference map respectively for molar class, (A3,4) SR result with scaling factor of 4 and difference map respectively for molar class. (B) Ground truth for premolar class, (B1,2) SR result with scaling factor of 2 and difference map respectively for premolar class, (B3,4) SR result with scaling factor of 4 and difference map respectively for premolar class. (C) Ground truth for canine class, (C1,2) SR result with scaling factor of 2 and difference map respectively for canine class, (C3,4) SR result with scaling factor of 4 and difference map respectively for canine class. (D) Ground truth for incisor class, (D1,2) SR result with scaling factor of 2 and difference map respectively for incisor class, (D3,4) SR result with scaling factor of 4 and difference map respectively for incisor class.

Qualitative results are also given in Figures 2–4 respectively to ESPCN, SRCNN, and ESRT. The visualization through difference maps illustrates the discrepancies between the generated image and the ground truth image for each models.

According to Figure 2, for SF of 2, ESPCN networks provide results closer to the ground-truth edges and details seem reasonably reconstructed. However, subtle artifacts or blurriness may be presents. Also difference map tends to show lower intensity overall, indicating smaller reconstruction errors. Moreover, errors are primarily concentrated around high-frequency regions like edges or fine details. For the SF of 4, ESPCN network demonstrates noticeable degradation in quality compared to the ground truth. Blurring and loss of details are more pronounced, particularly in areas requiring high-frequency detail reconstruction. Difference maps for the SF of 4 are significantly brighter, indicating larger discrepancies between the SR images and related ground truth.

**Figure 5.**
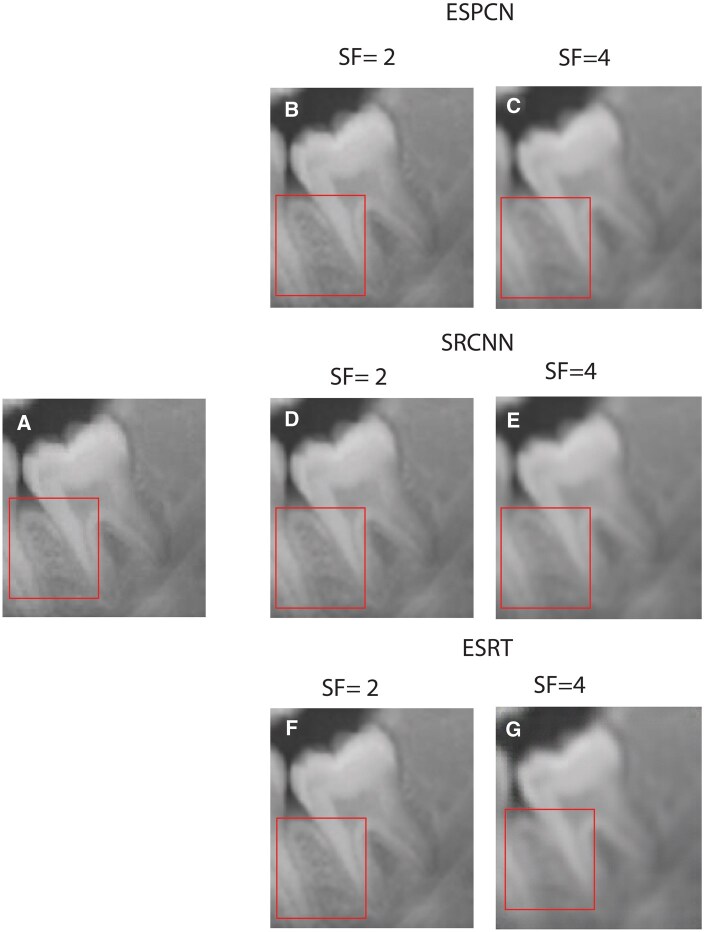
(A) Original image. (B) HR image with ESPCN network scaling factor (SF) of 2. (C) HR image with ESPCN network scaling factor (SF) of 4. (D) HR image with SRCNN network scaling factor (SF) of 2. (E) HR image with SRCNN network scaling factor (SF) of 4. (F) HR image with ESRT network scaling factor (SF) of 2. (G) HR image with ESRT network scaling factor (SF) of 4. Abbreviations: ESPCN = Efficient Subpixel Super Resolution; ESRT = Efficient Super-Resolution Transformer; SF = scaling factor; SRCNN = Super-Resolution Convolutional Neural Network.

**Figure 6.**
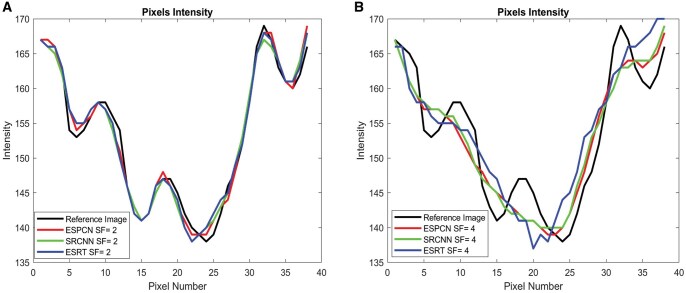
(A) Intensity plot of selected 1 line of pixel from red region for scaling factor of 2. (B) Intensity plot of selected 1 line of pixel from red region for scaling factor of 4. Abbreviations: ESPCN = Efficient Subpixel Super Resolution; ESRT = Efficient Super-Resolution Transformer; SF = scaling factor; SRCNN = Super-Resolution Convolutional Neural Network.

Figure 3, for the SF of 2, provides results closer to the ground truth in comparison to SF of 4. However, some fine details are still missing or slightly blurred. Compared to the ESPCN, the difference map has might be slightly brighter in some areas, indicating a slightly higher reconstruction error for the SF of 2. For SF of 4, the quality of the SR-images noticeably degrades and blurring and loss of details are evident particularly around high-frequency regions such as edge and fine texture. Figure 4 indicates that model performs well, with high SSIM and PSNR, preserving structural details with minimal degradation for SF = 2. However, SF = 4 results show significant quality loss, with SSIM dropping to approximately 0.82–0.88 and PSNR to 31–33 dB, leading to noticeable noise and blurriness. Molar images retain the highest quality, while premolars and incisors degrade more due to finer structures. For more clarity, acquired results from these networks are compared in Figure 5 and their intensity plots are given in Figure 6. From Figure 5, as the scaling ratio increased, the clarity of the borders in the images decreased and became smooth and then blurred-like.

The red-boxed areas contain fine dental structures that challenge the SR models. At SF = 2, the structural integrity is generally well-preserved across all models, with ESRT producing a balanced reconstruction that maintains texture sharpness without excessive artifacts. ESPCN introduces some artificial sharpening, while SRCNN appears slightly more blurred but maintains a smooth texture. At SF = 4, the quality degradation is evident—details within the red-boxed regions become less defined. ESRT, while still capturing the general structure, loses some sharpness, and ESPCN and SRCNN struggle with pixelation and texture distortion. This aligns with the intensity plots, where larger deviations in pixel intensity indicate a loss of finer details and inconsistencies in texture reproduction. The results suggest that at SF = 2, ESRT is effective in maintaining intensity variations and structural details, making it a strong candidate for moderate SR tasks. However, at SF = 4, all models struggle to reconstruct fine structures accurately, with noticeable differences in how they handle texture loss and intensity variations. ESRT’s tendency to overshoot in some regions suggests it attempts to retain high-frequency details but at the cost of some distortion, while ESPCN and SRCNN exhibit smoother, albeit less detailed, reconstructions. This highlights the challenge of extreme upscaling and the need for advanced techniques to preserve fine textures and structural integrity in medical imaging applications.

The intensity plots in Figure 5 set of images correspond to the red-boxed regions in the second set of images, which highlight the fine dental structures being analysed for SR accuracy. The plots compare pixel intensity variations along a selected line from the reference image (black) and the outputs of 3 SR models: ESPCN (red), SRCNN (green), and ESRT (blue) at SFs of 2 and 4. At SF = 2 (left plot), all models closely follow the reference intensity pattern, indicating that they successfully preserve structural details with minor deviations. However, ESRT appears to align more closely with the reference image, particularly in regions with rapid intensity changes, suggesting better feature retention. At SF = 4 (right plot), deviations from the reference become more apparent across all models, with ESPCN and SRCNN tending to smooth out sharp transitions, while ESRT exhibits occasional overshooting, indicating an attempt to maintain high-frequency details but at the risk of introducing slight distortions.

Classification of tooth types, namely incisor, canine, premolar, and molar, was performed twice before and after SR application. Images of test sets were also used to obtain first classification performances of the deep classification models (*n* = 400 for each class). Table 2 presented classification performance of 4 different classification models using original images without SR application.

**Table 2.** Classification results of original images using their original resolution and images downscaled by 2 and 4.

	Original images (with their original resolutions)			
	Accuracy	Recall	Precision	F1 score
**ResNet**	0.8800	0.8790	0.8810	0.8800
**VGG**	0.8600	0.8630	0.8600	0.8620
**DenseNet**	0.8400	0.8409	0.8404	0.8407
**EfficientNet**	0.7600	0.7700	0.7580	0.7660
**ViT**	0.2500	0.2500	0.0600	0.0900

	Images downscaled by scaling factor of 2				Images downscaled by scaling factor of 4			
	Accuracy	Recall	Precision	F1 score	Accuracy	Recall	Precision	F1 score
**ResNet**	0.7850	0.7859	0.7891	0.7875	0.6750	0.6738	0.6769	0.6754
**VGG**	0.8475	0.8499	0.8538	0.8518	0.8575	0.8563	0.8588	0.8576
**DenseNet**	0.8225	0.8244	0.8240	0.8242	0.7175	0.7167	0.7197	0.7182
**EfficientNet**	0.7025	0.7082	0.7011	0.7047	0.4650	0.4661	0.4674	0.4668

Model performances were evaluated by accuracy, recall, precision, and F1 score. Each metric was specified as the average of 4 classes. To have a benchmark for the effect of SR in classification tasks with SR generated images, classification was performed using original images that have original resolution. Classification using images that their resolution downscaled by 2 and 4 was provided for further analysis. It makes possible to compare classification performances using SR generated images with same resolution.

It was seen that the classification performance with the original resolution images showed different trends in different models. Except for VGG, the performance of the other 3 classification models decreased when the resolution was reduced by 2 and 4. While VGG showed a robust performance to the image resolution, it was observed that the EfficientNet model was very sensitive to the image resolution and its performance decreases more with decreasing resolution.

Followed by the first classification procedure, images generated by SR models were also classified to compare classification performance after SR application. Table 3 showed classification results of the images generated by ESPCN, SRCNN and ESRT networks.

**Table 3.** Classification results on super resolved images with ESPCN, SRCNN and ESRT networks with scaling factor of 2 and 4.

	Super resolution with scaling factor of 2 using ESPCN				Super resolution with scaling factor of 4 using ESPCN			
	Accuracy	Recall	Precision	F1 score	Accuracy	Recall	Precision	F1 score
**ResNet**	0.8250	0.8273	0.8333	0.8303	0.8525	0.8520	0.8548	0.8534
**VGG**	0.8575	0.8583	0.8589	0.8586	0.8175	0.8112	0.8129	0.8120
**DenseNet**	0.8750	0.8771	0.8773	0.8772	0.8475	0.8486	0.8481	0.8476
**EfficientNet**	0.8050	0.8052	0.8062	0.8057	0.7925	0.7977	0.7936	0.7957

	Super resolution with scaling factor of 2 using SRCNN				Super resolution with scaling factor of 4 using SRCNN			
	Accuracy	Recall	Precision	F1 score	Accuracy	Precision	Recall	F1 score
**ResNet**	0.8200	0.8198	0.8205	0.8202	0.7650	0.7644	0.7676	0.7660
**VGG**	0.8125	0.8149	0.8171	0.8160	0.7950	0.7971	0.7971	0.7971
**DenseNet**	0.8675	0.8670	0.8672	0.8671	**0.8525**	**0.8529**	**0.8509**	**0.8519**
**EfficientNet**	0.7925	0.7915	0.7922	0.7918	0.7825	0.7809	0.7859	0.7834

	Super resolution with scaling factor of 2 using ESRT				Super resolution with scaling factor of 4 using ESRT			
	Accuracy	Recall	Precision	F1 score	Accuracy	Precision	Recall	F1 score
**ResNet**	0.5525	0.5525	0.7105	0.5220	0.6200	0.6200	0.8135	0.5822
**VGG**	0.8375	0.8375	0.8402	0.8376	0.8600	0.8600	0.8633	0.8612
**DenseNet**	0.8350	0.8350	0.8438	0.8355	0.9225	0.9225	0.9231	0.9224
**EfficientNet**	0.7525	0.7525	0.7624	0.7463	0.8900	0.8900	0.9057	0.8861

Abbreviations: ESPCN = Efficient Subpixel Super Resolution; ESRT **=** Efficient Super-Resolution Transformer; SRCNN = Super-Resolution Convolutional Neural Network.

**Figure 7.**
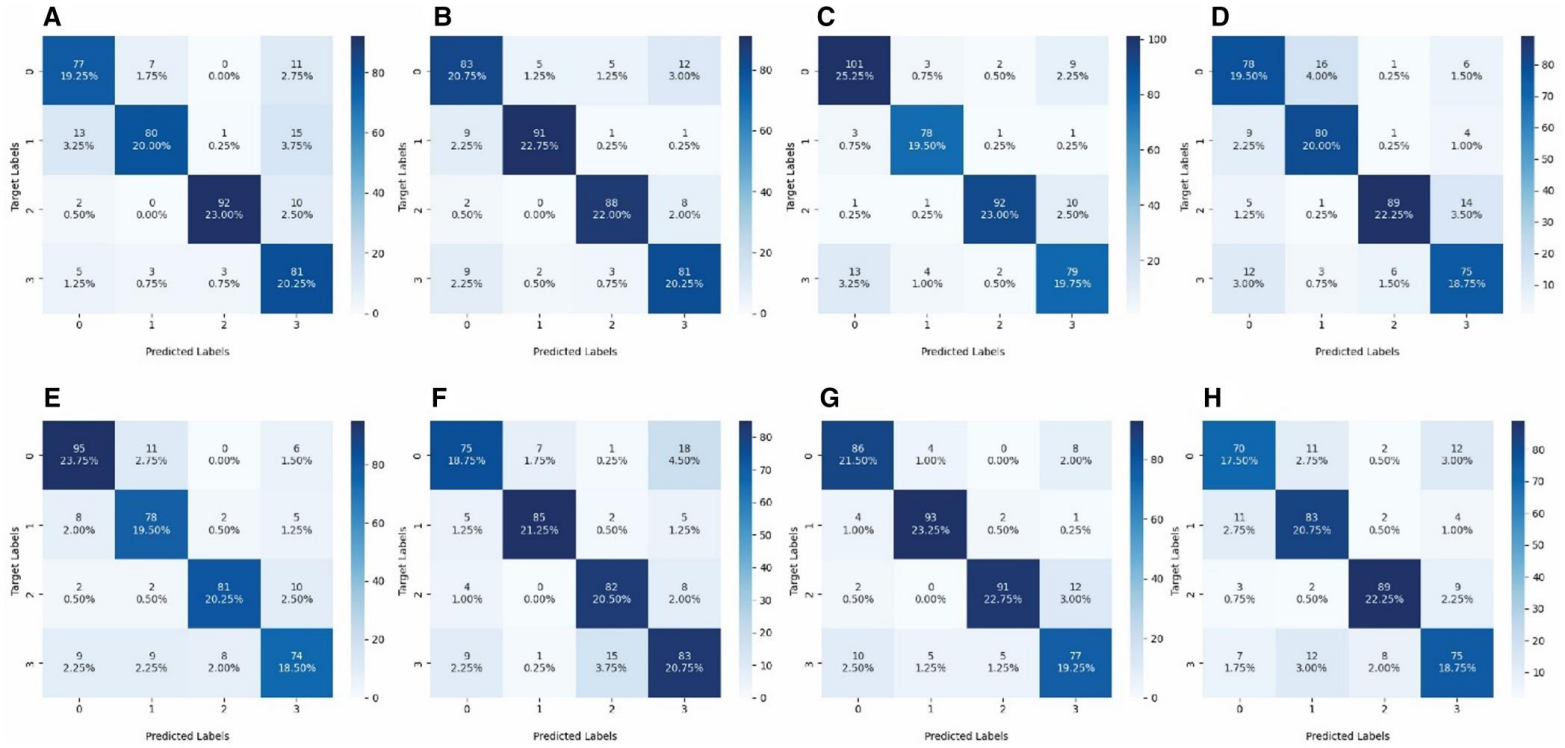
Confusion matrix of super resolved images with scaling factor of 2. (A) Super resolved with ESPCN and classified with ResNet50. (B) Super resolved with ESPCN and classified with VGG. (C) Super resolved with ESPCN and classified with DenseNet. (D) Super resolved with ESPCN and classified with EfficientNet. (E) Super resolved with SRCNN and classified with Resnet50. (F) Super resolved with SRCNN and classified with VGG. (G) Super resolved with SRCNN and classified with DenseNet. (H) Super resolved with SRCNN and classified with EfficientNet.

It was seen that SR remarkably improved classification performance for both SFs. ESPCN provided images that resulted in superior classification performance. Confusion matrix regarding classification task with SF of 2 was given in Figure 7.

When it comes to comparing classification performances for the situations with SR and without SR, there might be 2 approaches, as explained before in Figure 3. For Type-1, it was observed that DenseNet showed higher accuracy (0.875 vs 0.84) in the classification made by the ESPCN model with the images whose resolution was increased by a factor of 2 compared to the classification made with the original images, while the other 3 models showed similar or very close results. When the resolution was increased by a factor of 4, the EfficientNet model showed higher accuracy (0.79 vs 0.76), while the other 3 models produced similar results to the initial results. When the resolution was increased 2-fold using the SRCNN model, EfficientNet and DenseNet models increased the accuracy (0.792 vs 0.76–0.867 vs 0.84), while the other 2 models produced results close to the original classification. When the resolution was increased 4-fold, DenseNet and EfficientNet improved the accuracy performance compared to the original classification performance, but ResNet and VGG performance remained relatively lower. For Type-2, it was shown that classification performances were significantly improved by SR for almost all cases in terms of classification models and scaling ratios.

## Discussion

Trends in recent years have showed us that AI, especially deep learning, has emerged as a pivotal force in various branches of dentistry, demonstrating exceptional proficiency in data analysis, pattern recognition, and dental image analysis.^1–4^^,^^30–32^ The integration of these advanced algorithms into dentistry-based procedures has catalysed significant advancements, providing innovative solutions and enhancing traditional methods for diagnosis and treatment planning across dental specialties.

As an emerging and cutting-edge field of research, SR is a process in computer vision where an LR image is transformed into an accurate HR image by learning spatial hierarchies and extracting features from images. It is based on convolutional neural networks and generative adversarial networks. In dentistry, it is getting more attention day-by-day, thanks to its significant potential for helping dentists in diagnosis and treatment via improved visualization, increased clarity and detail.

The present work evaluates the effect of SR on the classification performance of teeth types. Teeth images from panoramic radiographs of an open access dental dataset, the Tufts dental database, are categorized into 4 classes, namely incisor, canine, premolar, and molar. Firstly, original images are classified using 4 deep learning models, ResNet50, VGG-19, DenseNet, and EfficientNet. Later, SR is applied by 2 different models, ESPCN and SRCNN, with scaling ratios of 2 and 4. Lastly, images generated by SR models are classified again using the same 4 classification models to determine the effect of SR. Findings showed that SR with both ESPCN and SRCNN improved classification performance of all classification models implemented. According to acquired results, SR has improved classification performance of all networks. However, the amount of this improvement is more discernable while SR performed with ESPCN network.

ESPCN and DenseNet perform better due to their complementary strengths in SR and classification tasks. ESPCN excels in reconstructing high-fidelity images by using sub-pixel convolution layers, which preserve spatial details and effectively learn high-frequency features, resulting in superior performance even for challenging SFs like SF = 4. DenseNet, on the other hand, leverages its dense connections to reuse features across layers, ensuring robust feature extraction, better gradient flow, and high accuracy in classification. On the other hand, ResNet shows the highest performance with original images due to its powerful residual learning framework, which enables the efficient extraction of high-level features while addressing the vanishing gradient problem. Its residual connections allow for effective training on deeper networks, making it highly suitable for handling the full-resolution, high-quality input provided by the original images. This advantage enables ResNet to achieve the highest accuracy, recall, precision, and F1 scores in tasks involving original images. However, after SR, ResNet’s performance drops to the third position because it may struggle to fully adapt to the reconstructed high-frequency details and potential artifacts introduced by the SR methods.

It was also seen that as the scaling ratio increased, the clarity of the borders in the images decreased and became smooth and then blurred-like. This is primarily due to the loss of high-frequency details that define sharp edges and borders. While scaling from an LR input, the model must generate additional pixel information, often leading to ambiguity in accurately reconstructing fine details, especially along edges. This ambiguity causes edges to become smoothed or blurred as the model struggles to differentiate borders from surrounding textures. Another contributing factor is the inherent limitations of the model’s training data and architecture. If the training dataset lacks sufficient examples of sharp transitions at high resolutions, the model may generalize poorly to such features. The evaluation of different SR models at SFs of 2 and 4 highlights the trade-offs between detail preservation and artifact introduction. According to the results, ESPCN and SRCNN perform reasonably well at SF = 2, producing images that closely resemble the ground truth with minimal blurriness and artifacts. However, as shown in the difference maps, reconstruction errors are still present, particularly around high-frequency regions like edges and textures. At SF = 4, a significant degradation in image quality is observed, with increased blurring and loss of fine details, particularly in regions requiring high-frequency reconstruction. The difference maps at SF = 4 further indicate larger discrepancies between the super-resolved images and the ground truth, suggesting that these models struggle to retain structural integrity at higher magnifications. Similarly, the intensity plots confirm that at SF = 2, the pixel intensity variations of ESPCN, SRCNN, and ESRT align well with the reference, whereas at SF = 4, deviations become more apparent, with ESPCN and SRCNN smoothing out transitions, while ESRT exhibits slight overshooting. On the other hand, the qualitative analysis from red-boxed regions further illustrates the challenges faced by these models in reconstructing fine dental structures. ESRT demonstrates a balanced approach at SF = 2, maintaining sharper textures with minimal distortions, whereas ESPCN introduces slight artificial sharpening, and SRCNN generates a smoother yet slightly blurred texture. At SF = 4, however, all models exhibit pronounced degradation, with ESRT attempting to retain high-frequency details but at the cost of overshooting, while ESPCN and SRCNN struggle with pixelation and texture distortions. This is evident in the intensity plots, where deviations from the reference become more noticeable at SF = 4, reinforcing the difficulty of preserving fine details under extreme upscaling.

Comparison of classification performances was categorized into 2 approaches, namely Type-1 and Type-2, as explained in Figure 3. Considering Type-1, almost half of the classification models provided superior accuracy compared to classification with original images. Type-2 refers to a situation that can be imagined in the future when AI solutions are improved and integrated into the system by addressing today’s limitations. In almost all cases, classification accuracy is higher with SR-generated images. This shows that dental radiographs whose resolution is increased with SR models offer higher performance in the classification of tooth types. On the other hand, it is also an indication that it will be an extremely useful and valuable tool in the future.

In dentistry, applications based on SR with deep learning models is limited. To the best of our knowledge, among the limited studies using SR with deep learning, no other study has examined the effect of SR on a classification task.

Previously, Hwang et al^33^ examined the use of deep learning to restore compressed CBCT images, addressing storage space and cost challenges due to large 3D imaging data in dentistry. Using a publicly available dataset, virtual CBCT images were created and a very deep SR (VDSR) network was trained to enhance LR CBCT images. The results showed that VDSR provided better image quality than traditional bicubic interpolation, with a clinically acceptable compression scale ratio of 2.1. The study concluded that VDSR offered promising restoration accuracy, with future work needed on new algorithms and larger datasets for clinical application.

Celik et al^18^ explored the use of deep learning-based SR techniques to enhance the quality of dental panoramic radiographs, which are often limited by poor resolution. Four models were evaluated—SRCNN, Efficient Sub-Pixel Convolutional Network, Super-Resolution Generative Adversarial Network, and Autoencoder—using 1714 radiographs from open datasets. The models reconstructed HR images from LR inputs at varying scales (2x, 4x, 8x). Results were measured with SSIM and PSNR, with SRCNN yielding the best performance.^18^

Rahimi et al compared deep learning-based SR models with a conventional method for improving the resolution of dental panoramic radiographs. A total of 888 radiographs were used, and 5 SR models—SRCNN, SRGAN, U-Net, SwinIr, and LTE—were tested against the traditional bicubic interpolation method. Performance was evaluated using MSE, PSNR, SSIM, and expert opinion (MOS). The LTE model performed best, with MSE of 7.42, SSIM of 0.919, PSNR of 39.74, and MOS of 3.59. All SR models significantly improved image quality compared to LR images, with LTE showing the highest overall performance.

Moran et al^13^ investigated the impact of resolution enhancement methods on oral imaging for periodontal disease assessment. Due to limitations in imaging tools, many oral examinations had low resolution, complicating lesion evaluation. The study compared traditional methods (nearest, bilinear, bicubic, Lanczos) and deep-learning approaches (SRCNN, SRGAN) for improving image resolution. In the first part, specialized dentists visually evaluated images processed with these methods. In the second part, these techniques were used as preprocessing steps for CNN classifiers (Inception and ResNet) to assess whether resolution enhancement improves classification performance. While deep-learning methods significantly enhanced image quality, they did not consistently improve classifier performance.

According to Kumar et al,^34^ some details, such as gums and mucous membrane, exact shape and contours of tooth surface, specific arrangement of teeth, and in paediatric dentistry, detailed development of particular teeth, are less significant in dental X-ray images. Applying SR methods could be helpful not only during diagnosis and treatment stages but also for automatic classification and detection of particular condition in dental X-rays by acknowledging the fact that providing images with higher resolution could provide more details of the dental images.

SR improves classification performance by enhancing image quality, making it particularly valuable in engineering and clinical applications. From an engineering perspective, SR reconstructs high-frequency details like edges and textures lost during image acquisition or downscaling, providing higher-quality inputs for classification models. Clinically, SR enhances diagnostic accuracy by improving the clarity of critical details, such as anomalies or textures, which are essential for reliable diagnoses. It facilitates expert validation by providing clearer visual information, enabling clinicians to cross-check AI-driven results. Furthermore, SR allows LR images, common due to hardware limitations or patient motion, to be effectively used in clinical workflows, supporting critical decision-making in time-sensitive scenarios. By bridging the gap between hardware constraints and the need for HR data, SR empowers more accurate and efficient decision-making in both engineering and clinical contexts. Despite the remarkable success of deep learning in various domains, several limitations hinder its broader applicability and reliability. One of the primary challenges is the need for large, multi-centred labelled datasets, as deep learning models often struggle with limited or imbalanced data. Moreover, there is not a consensus on reporting results that have the potential to improve benchmarking for the following works. In the future, SR-assisted image analysis systems could be integrated into various imaging modalities in clinical areas. With the capacity to create HR images from low-quality images, SR algorithms can provide cost-effective solutions for medical imaging devices and help overcome the limitations of existing hardware.

## Conclusion

The present work demonstrated that getting HR images via SR models can improve classification performance for tooth types using panoramic radiographs.
